# Combination of Aβ Secretion and Oxidative Stress in an Alzheimer-Like Cell Line Leads to the Over-Expression of the Nucleotide Excision Repair Proteins DDB2 and XPC

**DOI:** 10.3390/ijms160817422

**Published:** 2015-07-30

**Authors:** Anne Forestier, Thierry Douki, Viviana De Rosa, David Béal, Walid Rachidi

**Affiliations:** Laboratoire Lésions des Acides Nucléiques, Université Joseph Fourier-Grenoble 1/CEA/Institut Nanoscience et Cryogénie/SCIB, UMR-E3, Grenoble, France; E-Mails: ann.forestier@gmail.com (A.F.); thierry.douki@cea.fr (T.D.); viviana.derosa@ibb.cnr.it (V.D.R.); david.beal@cea.fr (D.B.)

**Keywords:** neurodegenerative disorders, Alzheimer’s disease, DNA damage, DNA repair, nucleotide excision repair, XPC, DDB2, oxidative stress

## Abstract

Repair of oxidative DNA damage, particularly Base Excision Repair (BER), impairment is often associated with Alzheimer’s disease pathology. Here, we aimed at investigating the complete Nucleotide Excision Repair (NER), a DNA repair pathway involved in the removal of bulky DNA adducts, status in an Alzheimer-like cell line. The level of DNA damage was quantified using mass spectrometry, NER gene expression was assessed by qPCR, and the NER protein activity was analysed through a modified version of the COMET assay. Interestingly, we found that in the presence of the Amyloid β peptide (Aβ), NER factors were upregulated at the mRNA level and that NER capacities were also specifically increased following oxidative stress. Surprisingly, NER capacities were not differentially improved following a typical NER-triggering of ultraviolet C (UVC) stress. Oxidative stress generates a differential and specific DNA damage response in the presence of Aβ. We hypothesized that the release of NER components such as DNA damage binding protein 2 (DDB2) and Xeroderma Pigmentosum complementation group C protein (XPC) following oxidative stress might putatively involve their apoptotic role rather than DNA repair function.

## 1. Introduction

Alzheimer’s disease (AD) is the most common neurodegenerative disease, which progressively leads to massive neuronal death. The physiopathology of AD is characterized by two pathological hallmarks: within neurons, the accumulation of hyperphosphorylated Tau protein leads to the formation of neurofibrillary tangles [1]; in the extraneuronal environment, the abnormal proteolytic processing of the amyloid precursor protein (APP) leads to the aggregation of senile plaques, or amyloid plaques. AD brains exhibit a massive apoptosis, which is known to be a cellular response to excess DNA damage that triggers a programmed cell death mechanism [2]. The link between AD and accumulation of DNA damage has led several groups to study the DNA repair capacities in AD patients, mouse models and cell lines and, as a general trend, a lower ability to maintain genomic integrity was observed [3,4,5,6,7,8,9,10,11].

Cells possess several DNA repair pathways, each of them is in charge of a specific class of lesions. The base excision repair (BER) pathway is preferentially involved in removing and replacing either methylated, oxidized or deaminated (uracil) bases and single strand breaks [12]. Nucleotide excision repair (NER) is predominantly involved in the repair of bulky adducts, such as UV-induced photoproducts, adducts to chemicals and DNA-DNA or DNA-protein crosslinks [13]. NER actually consists of two sub-pathways: Global Genome Repair (GG-NER) and Transcription-Coupled Repair (TC-NER). GG-NER surveys the whole genome while TC-NER removes RNA Polymerase-blocking lesions in transcribed strands of active genes [14]. Other pathways, homologous recombination (HR) and non-homologous end joining (NHEJ) are in charge of the repair of double strand breaks (DSB) [15].

However, the DNA repair can be impaired, and thus involved in many of pathologies. The most obvious consequence is an increased susceptibility to cancer because of accumulation of mutations. Yet, decreased DNA repair capacities are also involved in neurological disorders, as illustrated by a series of genetic diseases that impact the nucleotide excision repair. The best known example is Xeroderma Pigmentosum (XP) and its seven XPA to XPG subgroups corresponding to the associated mutated NER enzyme [16]. If the main feature of the XP disease is the high susceptibility to develop skin cancers, neurodegeneration could be observed in some cases (XPA, XPB, XPF, XPG) [17]. Cockayne’s Syndrome (CS), another autosomal recessive congenital disorder, characterized by mutations in transcription-coupled NER enzymes CSA and CSB, leads to a defective development of the central nervous system (CNS), a high sensitivity to sunlight and a premature aging [18]. Association of NER and Alzheimer’s disease has been poorly studied, while a few papers suggest that the mechanisms underlying neurological impairment in XP or CS could be similar to those involved in AD [19].

It may be added that although data have been gathered on the reduced DNA repair capacities in AD, no real mechanistic investigation has been made to explain this result. We previously showed that the secretion of the AD-specific neurotoxic peptide Aβ in a neuroblastoma cell line led to an overall decrease of BER, either at the basal level or following oxidative stress [20]. The present work aimed at extending the work to NER, first because of the link between neuronal disorders and deficiency in this repair pathway. In addition, oxidative stress not only induces breaks and oxidized damages but may also lead to bulky lesions that are likely substrates for NER. For this purpose, we studied the repair of pyrimidine dimeric photoproducts induced by UVC radiation and quantified the expression of a series of genes in an Alzheimer-like cell line. The obtained results led us to propose a role outside of repair in AD for some NER proteins.

## 2. Results

### 2.1. UVC-Induced Cytotoxicity Is Similar in Mock and APP751-Expressing Cells at Low Doses

**Figure 1:**
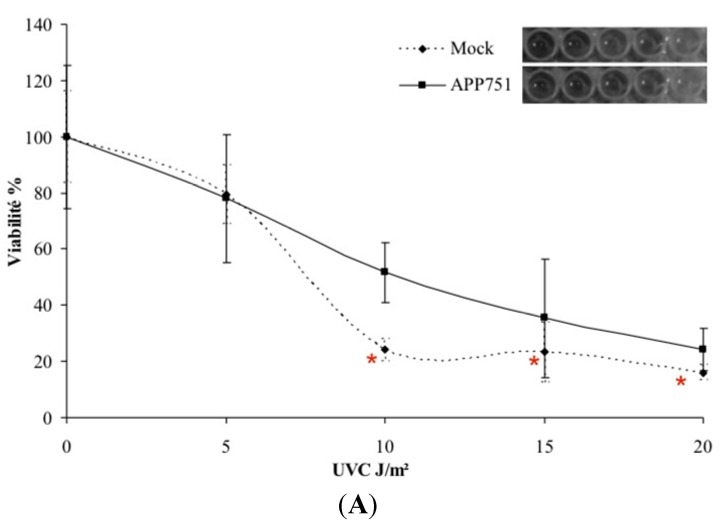

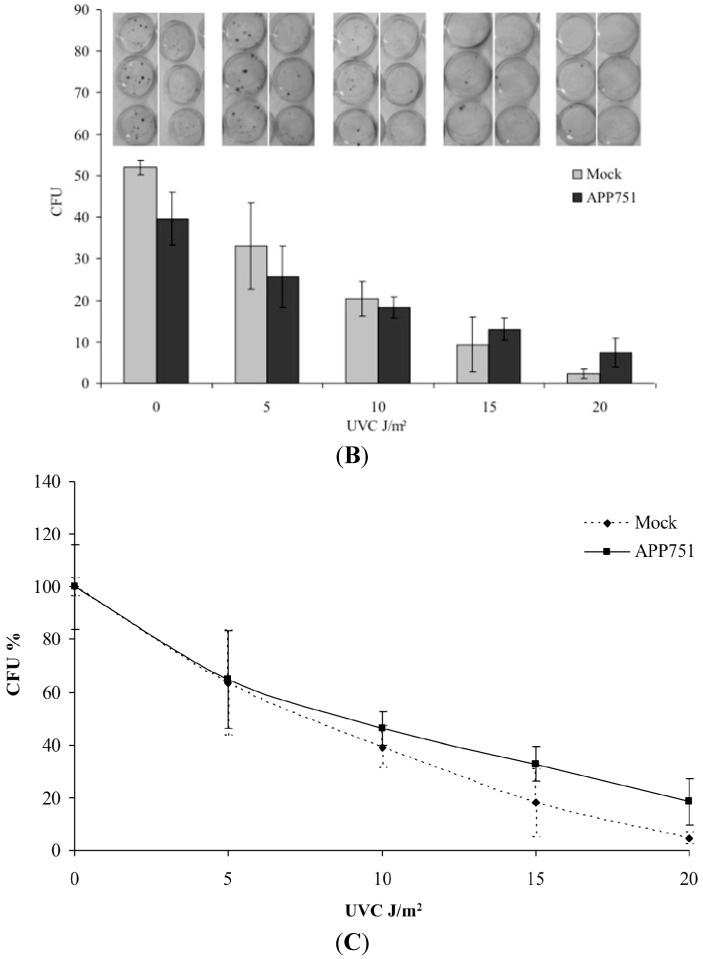
UVC cytotoxicity at short and long-term. Mock and APP751-expressing cells were cultured 48 h prior to irradiation. Then, four doses of UVC 5, 10, 15 and 20 J/m^2^ were tested. For short-term cytotoxicity, cells were grown for additional 24 h and then the MTT assay was performed to assess cell viability (**A**). Significance was assessed through the Student *t*-test (*n* = 3). * Data significantly different from mock cells (*p* < 0.05); For long-term toxicity, the cells were grown for an additional 12 days and then the colonies were revealed using crystal violet, and manually counted. The bar graph represents the number of colony forming units (CFU) in relation to the dose used; a representative picture of petri dishes following revelation with crystal violet is given above each bar of the graph, corresponding to each tested condition in triplicate (**B**); (**C**) represents the evolution of CFU in percentage Significance assessed through the Student *t*-test (*n* = 3).

To assess short and long-term toxicity following exposure to UVC, we used the MTT (Figure 1A) and clonogenicity (Figure 1B,C) assays. Four doses of UVC irradiation were tested. At 5 J/cm^2^, the mock and APP751 cell lines exhibited similar viability (79.4% *versus* 78% respectively). At the same dose, the clonogenic potential was 63.5% for mock and 64.7% for APP751. At 10 J/cm^2^, the mock cell line appeared to be more sensitive to UVC irradiation with a survival of 24% against 51.8% for the APP751-expressing cells (*p* = 0.0001). However, the 10 J/cm^2^-associated clonogenic potential was similar in the two cell lines (39.1% for mock and 46.2% for APP751). After 15 J/cm^2^ irradiation the mock viability was more affected (23.3% left) than the APP751-expressing cells (35.4%, *p* = 0.04) although the clonogenic potential was not significantly different between mock and APP751 (17.9% and 32.7%, respectively). At 20 J/cm^2^, the mock cell line was once again more sensitive to irradiation than the APP751 cells with a residual survival of 16.1% and 24.2% respectively (*p* = 4 × 10^−5^). Clonogenic potential was not more affected in one cell line compared to the other. As the lowest irradiation dose (5 J/cm^2^) did not induce significant difference in either MTT or *in vitro* colony forming assays between the two cell lines, it was chosen for further studies.

### 2.2. Effect of Aβ, H_2_O_2_ Treatment, and UVC Radiation on the NER-Associated Gene Expression in Mock and APP751-Expressing Cells

#### 2.2.1. Basal NER-Associated Gene Expression in the Mock and APP751-Expression Cells

**Figure 2:**
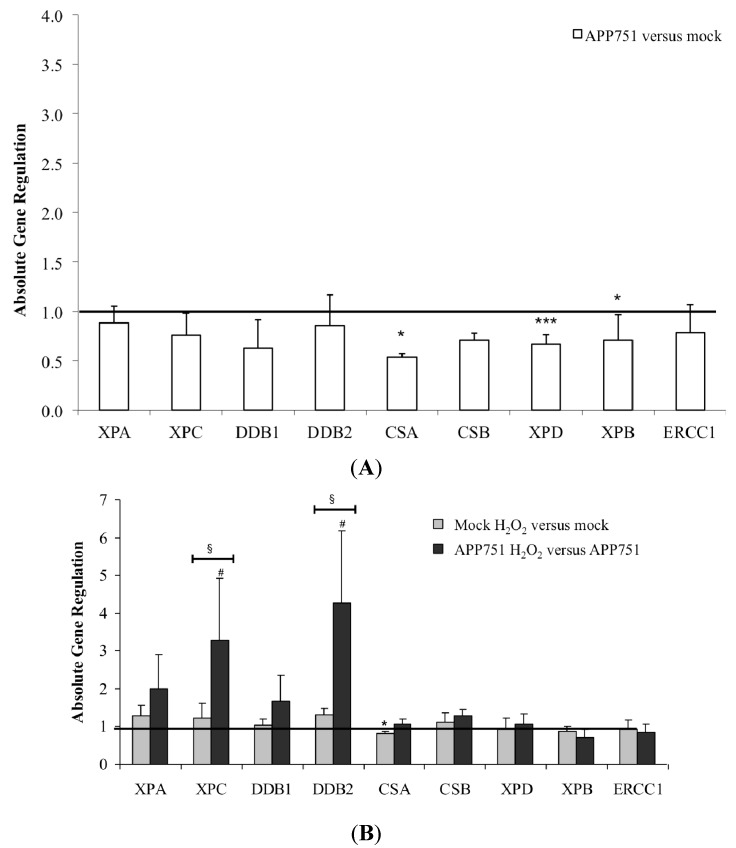

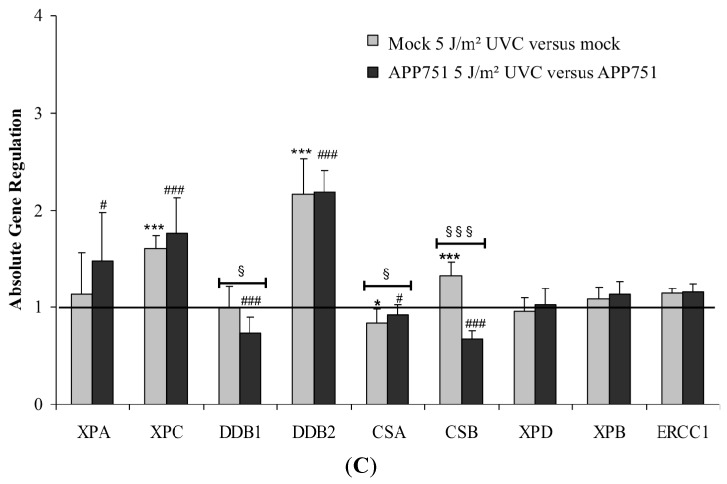
Aβ, H_2_O_2_, and UVC-induced NER gene expression. Mock and APP751-expressing cell lines were cultured for 48 h and left untreated (**A**) or H_2_O_2_-treated (115 µM) (**B**) or UVC-irradiated (5 J/m^2^) (**C**). Total RNA was extracted and subsequently reverse-transcribed. A total of 20 ng of corresponding cDNA were used to detect specific gene expression using real-time qPCR. Gene expression was investigated in APP751-expressing *versus* mock cells (**A**, *n* = 7), H_2_O_2_-treated cells *versus* untreated cells (**B**, *n* = 3) or UVC-irradiated cells *versus* untreated cells (**C**, *n* = 3). The mean of the corresponding expression ratios was calculated, and a Student’s *t*-test was performed. * data significantly different (*p* < 0.05) from mock cells, *** *p* < 0.0005; ^#^ data significantly different (*p* < 0.05) from APP751-expressing cells, ^###^ *p* < 0.0005 and ^§^ data significantly different (*p* < 0.05) from each other, ^§§§^ *p* < 0.0005.

The expression levels of DNA repair enzymes were measured using real-time quantitative PCR, which allows for the relative quantification of the expression of a target gene under one condition compared to another by normalizing to one or more housekeeping genes that are considered stably expressed in both conditions. We first investigated the expression level of NER-associated genes between the mock and the APP751-expressing cell lines under basal conditions without any exogenous stress (Figure 2A). The expression ratio of XPA, XPC, DDB1, DDB2, CSB and ERCC1 mRNA levels were not significantly different between the two cell lines. In contrast, the expression ratio of CSA was lower in the APP751-expresing cells compared to the mock ones (0.54 ± 0.04, *p* = 0.04). The fold change in expression in APP751-expressing cells compared to mock-transfected cells for XPD was 0.67 ± 0.19 (*p* = 0.0007). The expression of XPB changed 0.71 ± 0.26-fold in APP751-expressing cells compared to mock-transfected cells (*p* = 0.02).

Under basal condition (without any exogenous stress), the expression of most NER-associated genes was not significantly different between mock and APP751-expressing cells.

#### 2.2.2. Effect of H_2_O_2_ Treatment on NER-Associated Gene Expression in Mock and APP751 Cell Lines

We then examined the gene expression profile of the two cell lines following H_2_O_2_-induced oxidative stress (Figure 2B). The expression profiles of XPA, DDB1, XPD, XPB, CSB and ERCC1 were not significantly modified in both cell lines. In contrast, the expression ratio of CSA was diminished within the mock cell line following H_2_O_2_ exposure (0.83 ± 0.04, *p* = 0.02). After H_2_O_2_ treatment, XPC was not significantly modulated in the mock cell line, while it was over-expressed by the APP751 cell line (1.99 ± 0.92, *p* = 0.03). This difference in XPC expression was not only observed when comparing one treated cell line with its own control but also when comparing the two treated cell lines (*p* = 0.05). DDB2 mRNA level was slightly but significantly upregulated in the mock cell line (1.31 ± 0.15, *p* = 0.01). This trend was much more pronounced in the APP751-expressing cell line (4.28 ± 1.91, *p* = 0.02) after H_2_O_2_-induced oxidative stress. These expression ratios were also significantly different when the two treated cell lines were compared (*p* = 0.03).

Following H_2_O_2_ treatment, the expression profile was significantly different for several NER factors between the two cell lines. As the most salient features, XPC and DDB2 were upregulated in APP751-expressing cells compared to mock one, indicating an Aβ-dependent activation of these two factors following H_2_O_2_ treatment.

#### 2.2.3. Effect of UVC Radiation on the NER-Associated Gene Expression in Mock and APP751-Expressing Cells

Next, we compared NER gene expression profiles between the two cell lines following exposure to 5 J/m^2^ of UVC radiation (Figure 2C). XPA mRNA expression levels were not significantly modified in the mock cell line following irradiation, while it was upregulated in APP751-expressing cells (1.48 ± 0.49, *p* = 0.03). However, these ratios were not significantly different when the two irradiated cell lines were compared. XPC was significantly over-expressed in both mock and APP751 cell lines (1.61 ± 0.13, *p* = 1 × 10^−9^ and 1.76 ± 0.36, *p* = 0005 respectively) and these ratios were not significantly different from each other. The same scheme was observed for DDB2 with an increase in expression of 2.15 ± 0.37-fold for the mock cell line (*p* = 2 × 10^−7^) and of 2.18 ± 0.22-fold for the APP751 cell line (*p* = 1 × 10^−6^), these two UVC-induced upregulation being not significantly different from each other. Expression of DDB1 was not modified after UVC-irradiation in the mock cell line but it was significantly downregulated (0.73 ± 0.17, *p* = 0.002) in the APP751-expresing cell line. UVC irradiation did not induce any modulation within the mock or APP751-expressing cells, and accordingly no differences between the two cell lines for XPD, XPB or ERCC1. However, the expression ratio of CSA was reduced following UVC irradiation within both mock and APP751-expressing cells (0.84 ± 0.14, *p* = 0.05; 0.92 ± 0.10, *p* = 0.03, respectively). CSB was upregulated within the mock cells (1.32 ± 0.14, *p* = 2 × 10^−5^) and downregulated within the APP751-expressing cells (0.67 ± 0.08, *p* = 6 × 10^−8^).

The expression profile following UVC-induced stress was not different between mock and APP751-expressing cells. XPC and DDB2 were both over-expressed under the same extent, indicating that Aβ does not influence the activation of these two factors following UVC exposure.

### 2.3. H_2_O_2_ Treatment Specifically Enhances Excision Capacity of UVC Lesions by the APP751 Cell Line

**Figure 3:**
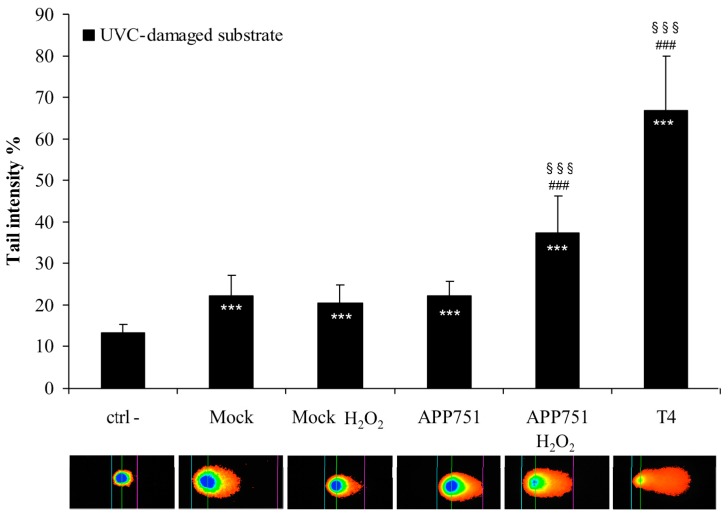
Analysis of photoproducts excision activity after H_2_O_2_ treatment. Mock and APP751-expressing cells were cultured for 48 h and either left untreated or incubated with H_2_O_2_ (115 µM) in the cell culture media for an additional 24 h. Total cell extracts were generated, and photoproducts excision capacity was analyzed on damaged genomic DNA substrates for 30 min using a modified version of the comet assay. The excision capacity of cell extracts was analyzed as the mean tail intensity of the comet. Three biological replicates were tested in triplicate in three independent experiments. The mean tail intensity of each cell extract (*n* = 3) was calculated and then a Student’s *t*-test was performed. Ctrl-: negative control (corresponds to the reaction buffer alone). T4: T4-endonuclease, positive control for the excision of the UV-induced DNA damage; *^#§^ data significantly different from Ctrl-, *** (*p* = 0.0005); ^###^ (*p* = 0.0005); ^§§§^ (*p* = 0.0005).

We used a comet-based assay to determine the efficiency of the NER system in our cell lines following H_2_O_2_ treatment. In this approach, UVC-irradiated nuclei from cells, used as substrate are incubated with protein extracts of the cells of interest. Excision of photoproducts by NER proteins induces additional breaks revealed by the Comet analysis (Figure 3). We first checked that the excision capacity of our cell extracts was significantly higher than the excision capacity of reaction buffer alone. Actually, the mean tail intensity of the UVC-damaged genomic substrates after exposure to cellular extracts was significantly higher than the tail intensity after incubation with reaction buffer alone, depicting a damage-specific incision by the cell extracts. We then showed that H_2_O_2_ treatment did not induce an increase in excision capacity within the mock cell line (22.35% for the control and 20.49% tail intensity after H_2_O_2_ treatment). In contrast, we observed that the excision capacity of the APP751 cell line that corresponds to 22.30% tail intensity under basal conditions increased to 37.45% (*p* = 3 × 10^−6^) after exposure to H_2_O_2_.

Thus, the photoproducts excision activity of cell extracts following H_2_O_2_-induced stress seems to be Aβ-dependent.

### 2.4. UVC Irradiation Induces a Similar Increase of Excision Capacity of UVC Lesions in Both Mock and APP751 Cell Lines

**Figure 4:**
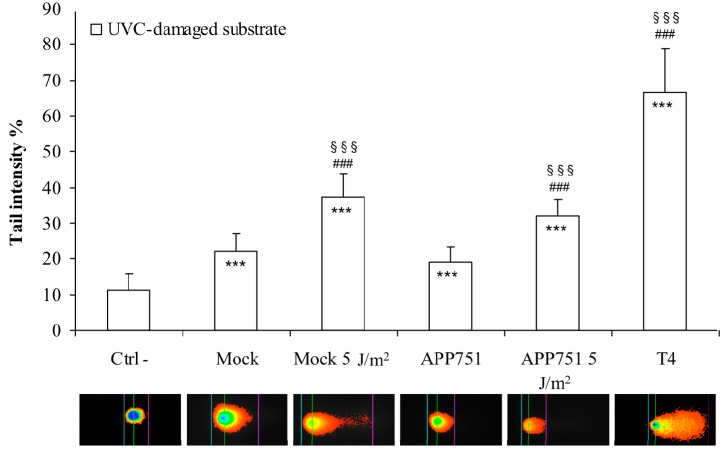
Analysis of photoproducts excision activity after UVC irradiation. Mock and APP751-expressing cells were cultured for 48 h and either left untreated or UVC-irradiated. Total cell extracts were generated, and photoproducts excision capacity was analyzed on damaged genomic DNA substrates for 30 min using a modified version of the comet assay. The excision capacity of cell extracts was analyzed as the mean tail intensity of the comet. Three biological replicates were tested in triplicate in three independent experiments. The mean tail intensity of each cell extract (*n* = 3) was calculated and then a Student’s *t*-test was performed. Ctrl-corresponds to reaction buffer alone. T4: T4-endonuclease, positive control for the excision of the UV-induced DNA damage; *^#§^ data significantly different from ctrl-, *** (*p* = 0.0005); ^###^ (*p* = 0.0005); ^§§§^ (*p* = 0.0005).

We further wanted to assess the excision capacity on UVC-damaged substrate of our cell extracts, following UVC irradiation (Figure 4). The excision capacity was significantly increased in both mock (from 22.15% under basal conditions to 37.22% tail intensity after irradiation, *p* = 0.0004) and APP751-expressing cells (from 19.26% to 31.92% tail intensity, *p* = 0.0001). No significant difference in terms of excision capacity was found between UVC-irradiated mock and UVC-irradiated APP751 cells.

The photoproducts excision capacity following UVC-irradiation is not influenced by Aβ.

### 2.5. Assessments of the Photoproducts Repair Kinetics in the Mock and APP751-Expressing Cells with or without H_2_O_2_ Pre-Treatment

**Figure 5:**
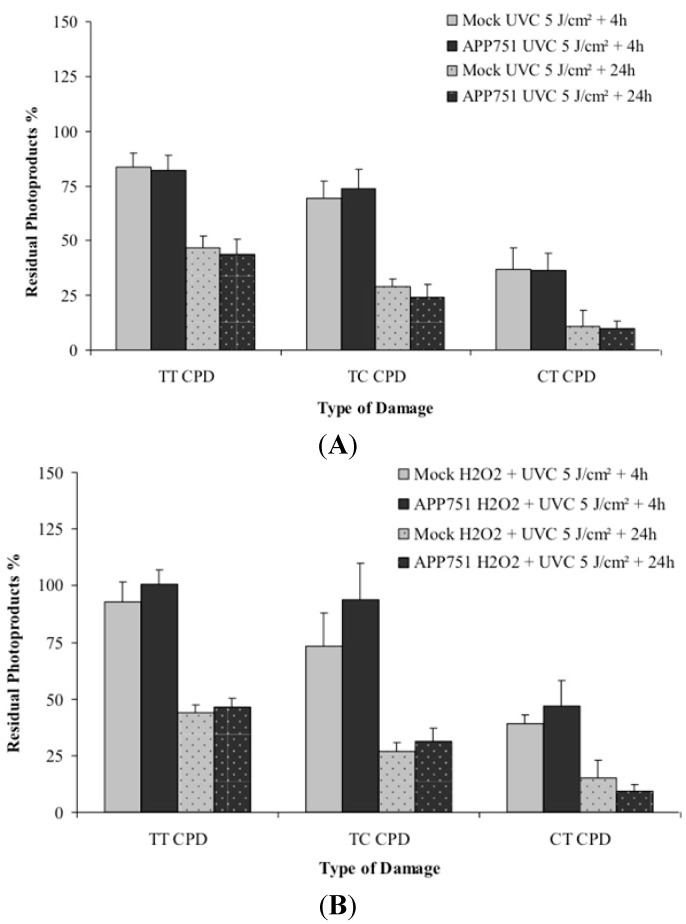
Assessments of the photoproducts repair kinetics in the mock and APP751-expressing cells. Persistence of cyclobutane dimers within Mock and APP751-expressing cells exposed to UVC (5 J/m^2^) radiation without (**A**) or with H_2_O_2_ pre-treatment (**B**). Repair of TT CPD, TC CPD and CT CPD are shown. The results are expressed in percentage of residual lesions and are the average ± SD of data obtained with three experiments.

We first wanted to assess the repair kinetics of cyclobutane pyrimidine dimers in our two cell lines, following 5 J/m^2^ UVC irradiation. Four hours after irradiation, the residual photoproducts percentage was not significantly different between the mock and APP751-expressing cells (Figure 5A): the level of thymine-thymine Cyclobutane Pyrimidine Dimers TT-CPD was 84% for mock and 82% for APP751; residual thymine-cytosine CPD (TC-CPD) was 69% for mock and 74% for APP751; and finally residual cytosine-thymine CPD (CT-CPD) was 37% for mock and 36% for APP751-expressing cells. Twenty-four hours after UVC irradiation, the level of residual CPD was also similar in the two cell lines. For mock and APP751 respectively, the level of TT-CPD was 47% and 44%, TC-CPD 29% and 24% and then CT-CPD 11% and 10%.

We then wanted to assess whether a H_2_O_2_ exposure prior to UVC irradiation would have an effect on CPD repair kinetics (Figure 5B). Four hours after irradiation, the level of any CPD was not significantly different between the two cell lines. The residual TT-CPD was 93% *versus* 101%, TC-CPD 73% *versus* 94% and CT-CPD 39% *versus* 47% for mock *versus* APP751-expressing cells respectively. Twenty-four hours after UVC irradiation, the level of the residual photoproducts was not either significantly different between the two cell lines. The residual TT-CPD level was 44% for mock and 46% for APP751, for TC-CPD it was 27% for mock and 31% for APP751 and finally for CT-CPD it was 15% for mock and 9% for APP751.

These results indicate that even though H_2_O_2_ treatment was shown to stimulate DDB2 and XPC gene expression, pre-incubating cells with H_2_O_2_ prior to UVC exposure does not influence the UVC-damage repair kinetics.

## 3. Discussion

Alzheimer’s disease is characterized by two major protein abnormalities. One of them involves the hyperphosphorylation of the microtubule-associated Tau protein and the other is the production and extracellular aggregation of the neurotoxic peptide Aβ [1].

DNA repair impairment is involved in both carcinogenesis and neurodegenerative disorders [21]. Given the high oxygen consumption of the brain, the high concentrations of polyunsaturated fatty acids that are highly susceptible to lipid peroxidation, its elevated content in redox active metals and the capacity of Aβ to generate reactive oxygen species, AD neurons are especially sensitive to oxidative stress [22]. As a matter of fact, these AD neurons exhibit increased oxidative damage to DNA [23]. ROS can lead to three type of DNA damage: small damaged bases, bulky helix distorting adducts and strand breaks [24]. A growing number of publications reveal that the small damaged bases and single strand breaks-associated DNA repair pathway, BER, is strongly diminished in the presence of Aβ although oxidative DNA damage was elevated [5]. While BER has been quite widely investigated in AD-associated studies, NER, in contrast, has been poorly analysed though its impairment could play a major role in the disease. Although BER and NER have been considered as harshly distinct and isolated pathways for a long time, this consensus has evolved over the past few years. Actually, numerous studies revealed that the compartmentalization of these two excision/synthesis pathways might not be so strict. A few oxidative lesions, mostly repaired by BER, are also repaired by XP proteins [25]. XPC (in addition to its role in NER pathway) could play an important role against oxidative stress. In favour of a protective role of XPC against oxidative DNA damage, it has been shown that XPC cells are hypersensitive to the killing effects of DNA-oxidizing agents due to the accumulation of oxidative DNA damage [26]. The protective function of XPC has been ascribed to its ability to affect the key BER enzymes such as thymine DNA and 8-oxoguanine DNA glycosylases. An *in vitro* study showed that 8oxoG-bearing oligonucleotides undergo an incision through a reconstituted NER system [27]. According to Yang [28], the interplay between NER and BER is not a matter of competition but rather of complementary and cooperative actions. Furthermore, the 8oxoG-specific glycosylase OGG1 activity is stimulated by the XPC/hHR23B complex [26,29]. In XPA-deficient cells, H_2_O_2_-induced DNA damage is repaired less efficiently than in normal cells [30]. CS proteins are also involved in oxidative stress-induced DNA damage. Cells from CS patients are extremely sensitive to oxidative stress [31,32,33], indicating that CSA or CSB-deficient cells are unable to repair oxidative damage.

The involvement of NER factors in the repair of oxidative damage constitute a hypothesis that could explain the phenomena of neurodegeneration in DNA repair-associated hereditary diseases such as Xeroderma Pigmentosum and Cockayne Syndrome [34]. By extension, NER impairment could also be involved in Alzheimer’s disease.

In our study we used two neuroblastoma cell lines either transfected with the pcDNA3 plasmid alone (mock cell line), or bearing the APP751 transgene (APP751-expressing cell line). The APP751 mutation corresponds to a double missense mutation, which was first highlighted in a Swedish family, and results in an increased secretion of Aβ at physiologically relevant levels.

We first compared NER-associated gene expression under basal conditions, without any exogenous stress, between the mock and APP751-expresing cell line. We observed that XPD and XPB were down regulated in the APP751-expressing cells compared to the mock cell line. This first observation counteracts previous published data indicating that the protein expression of these two helicases is increased in AD-affected human brains [35]. This difference in findings could be explained by the time of Aβ exposition undergone by the two distinct analysed samples. In Hermon’s study the samples came from AD patients whose neurons suffered the deleterious effects of Aβ for years. In our study, the APP751-expressing cells had been exposed to Aβ for only 48 h. As it is underlined by many publications, reduced DNA repair might be an early event in the etiology of AD [19] which correlates the mRNA down-regulation of XPB and XPD we observed. Over a certain threshold of DNA damage, as in Hermon’s study, the cell might set off a last rescue attempt to repair its DNA, which could explain the protein over-expression of XPD and XPB. More likely, these two helicases might not be involved in NER but in p53-mediated apoptosis as it was previously shown [36]. In APP751-expressing cells compared to mock under basal conditions, CSA mRNA expression was decreased. As we mentioned previously, a deficiency in CSA or CSB increases cell sensitivity to oxidative stress.

We then applied an oxidative stress and compared the resulting modulation of NER gene expression in our two cell lines. As anticipated, the mock cell line did not modulate NER gene expression after H_2_O_2_ exposition because oxidative DNA lesions mostly consist in small base damage and single strand breaks. Unexpectedly, the APP751-expressing cell line over-expressed genes like XPC or DDB2, which are associated with the repair of UV-induced damage by GG-NER. In order to gain further insights in the role of NER, we investigated the effect of a stress leading to DNA damage handled by the repair pathway, and chose UVC. Although not biologically relevant, UVC has the great advantage of avoiding addition of chemicals to damage DNA and of corresponding to very short treatment with minimal handling of the cells. So, we exposed the mock and APP751 cell lines to UVC and analysed the expression of the same set of NER genes as following H_2_O_2_-treatment. We showed that the two cell lines exhibited a similar modulation of the expression pattern upon UVC-irradiation with overexpression of the same NER genes, especially XPC and DDB2.

We then wanted to assess whether the differences observed in gene expression profiles was also encountered at the protein activity level. Thus we used a comet-based assay, allowing the quantification of the excision capacity of cell extracts, on UVC-induced damaged genomic substrate. When analysing H_2_O_2_-treated cells, we found no increase in excision capacity between untreated mock and H_2_O_2_-treated mock. On the contrary, exposure to H_2_O_2_ lead to a significant increase within the APP751-expressing cells, depicting the fact that the overexpression of NER genes such as XPC and DDB2 following an oxidative stress helped the Aβ-secreting cell line to enhance its capacity to excise UVC-induced bulky lesions. We then compared the evolution of UVC-induced damage excision capacity between the two cell lines following UVC irradiation. Here we found the same increase in excision capacity in both mock and APP751-expressing cells, data consistent with the same NER gene expression patterns. These latter results suggest that an oxidative stress could specifically enhance NER capacities through DDB2 and XPC over-expression.

For a better understanding of our results, we then hypothesized that exposure of cells to H_2_O_2_ prior to UVC irradiation could possibly stimulate APP751-cells and make them more competent for repairing UVC damage, as H_2_O_2_ treatment conferred upon them an enhanced photoproduct excision capacity according to the comet-based assay. This time, we used mass spectrometry lesion detection to perform a kinetic measurement of three photoproducts (TT-CPD, TC-CPD and CT-CPD). Unexpectedly, we found no significant differences in any of the CPD repair velocities, between the mock and APP751-expressing cells. It should be noted however that UVC increases expression of NER genes in the two cell lines. Thus, the specific effect of H_2_O_2_ on the repair of CPDs in APP751 cells could have been overwhelmed by the non-specific induction of BER by UVC radiation.

A first examination of these results could lead to the conclusion that the presence of Aβ leads to an increase in NER activity upon oxidative stress and thus may be somewhat protective. As we already mentioned, some NER factors are involved in the repair of small oxidative DNA damage usually repaired by BER. The mRNA upregulation we observed might also possibly be due to the recognition of another kind of oxidative damage. Indeed, even if they constitute only a small fraction of ROS-induced DNA damage, oxidative bulky adducts have been identified in model systems and isolated DNA and could be tremendously deleterious to the cell because they are more likely to directly trigger apoptosis than small base damage [37]. To explain the phenomenon of neurodegeneration observed in XP and CS patients, Robbins hypothesized that a hypothetic bulky lesion might occur in neurons, a cell type undergoing extensive oxidative stress. Without efficient NER, these hypothetic DNA damage may accumulate in neurons and lead to apoptosis [38]. Cyclopurines and tandem lesions could match the position of the hypothetic DNA damage, the latter being moreover weakly repaired by BER [39]. Another kind of bulky lesions could result from the addition of the breakdown products of lipid peroxidation such as 4-hydroxynonenal (HNE), another consequence of the oxidative stress triggered by Aβ [1]. It is important to note that lipid peroxidation occurs as an early event in the brain but also in peripheral tissues of AD patients [40].

Yet, this improvement of NER upon oxidative stress in the presence of Aβ is not completely satisfactory as an explanation to our observations following H_2_O_2_-treatment of APP751 cell lines. Indeed, an increase in NER pathway results in overexpression of a wider array of genes, as illustrated in the case of UVC irradiation. In contrast, only DDB2 and XPC are concerned by the effect of H_2_O_2_. Consequently, only the GGR part of NER is involved in this response, as seen in the activity measurements based on Comet assay. Indeed, DDB2 and XPC are DNA damage sensors in this repair sub-pathway. Yet this improved GGR activity in H_2_O_2_-treated APP751 cells is lower than that induced by the bulky pyrimidine dimers resulting from UVC irradiation as shown by the *in cellulo* measurements of CPD in combined H_2_O_2_/UVC experiments. It may be added that global genome repair is not an important mechanism in non-dividing cells like neurons. Accumulation of damage is mostly a concern in transcribed genes where it may inhibit synthesis of proteins or lead to impaired functionality. Accordingly, most links between DNA repair and neurodegenerative diseases are related to TCR [41].

This discussion invites us to look at the other putative roles of DDB2 and XPC. These proteins are mostly known for their participation in NER, as UVC-induced damaged sensors [42] but a growing number of publications suggest a role in DNA damage-induced apoptosis, not necessarily following a UV stress (reviewed in [43]). Actually, Stoyanova *et al.* [44] showed that DDB2 was required for DNA damage-induced apoptosis, following exposure to DNA damaging agents such as cisplatin [45]. Another study revealed that DDB2 over-expression within a cancer cell line led to higher cisplatin sensitivity through an increase in apoptosis [46]. Recently, Stergiou analysed the effects of an uncharacterized XPC mutation in *C. elegans* and showed that its capacity to trigger apoptosis following a genotoxic stress was reduced [47]. In our study, DDB2 and XPC could be specifically upregulated in the Aβ-secreting cell line, in response to the H_2_O_2_-induced DNA damage. It may thus be proposed that this process results in increased apoptosis. Indeed, Bagchi and Raychauduri [43] proposed that DDB2 could be a sensor of unrepaired DNA damage when their level is too high compared to the repair capacity of the cell. Finally, an interesting and very recent study demonstrates that p53-inducible DDB2 promotes apoptosis by mediating p21 degradation after ultraviolet UV-induced DNA damage [48]. As a matter of fact, neurons in AD exhibit a large amount of accumulated and unrepaired DNA damage over time [49,50], and DDB2 and/or XPC, through their sensor activity might further drive neurons into apoptosis.

## 4. Experimental Section

### 4.1. Cell Lines and Culture Conditions

The SK-SHSY5Y neuroblastoma cell line was either transfected with the pcDNA3 plasmid alone (mock cell line) or with pcDNA3 bearing the transgene corresponding to the Swedish mutation of APP (APP751-expressing cell line). The two cell lines were a gift from Luc Buée [51,52]. The cells were grown in Dulbecco’s modified Eagle’s medium/Glutamax™ supplemented with 10% fetal calf serum (FCS), 1 mM non-essential amino acids, 1% penicillin/streptomycin (Invitrogen, Saint-Aubin, France) and 400 µg/mL G418 (selection for cells expressing APP751 or the mock vector) in a 5% CO_2_ humidified incubator at 37 °C.

### 4.2. UVC-Induced Cytotoxicity Measurements

The mock and APP751-expressing cell lines were plated in 60 mm Petri dishes in 4 mL of medium and incubated at 37 °C for 48 h. The medium was removed, kept in falcons (Beckton Dickinson, POnt-De-Claix, France) and replaced by PBS containing Ca^2+^ and Mg^2+^ (Sigma, Saint-Quentin-Fallavier, France). Petri dishes were irradiated in triplicate at 5, 10, 15 or 20 J/m^2^ of UVC radiations (254 nm). A triplicate of non-irradiated dishes was also used as a control. PBS was then removed and the conditioned medium redistributed in its appropriated dish. Cells were then incubated for another 24 h at 37 °C. At the end of the incubation, cytotoxicity was assessed using a modified MTT (Sigma) assay. Briefly, 400 µL of MTT (5 mg/mL) in phosphate-buffered saline (PBS, Invitrogen) was added to each well. The medium was removed 2 h later, 3 mL of DMSO (Sigma) was added to dissolve the produced formazan, and the absorbance was read at 565 nm using a Multiskan RC microplate spectrophotometer (Labsystems, Nantes, France). The absorbance at 565 nm was proportional to the number of viable cells, and survival was calculated as the percentage of specific viability.

### 4.3. Long-Term Toxicity of UVC

To study the long-term toxicity of UVC, we performed an *in vitro* colony-forming assay, which is an *in vitro* cell survival assay, based on the ability of a single cell to grow into a colony after 2 weeks. Mock and APP751-expressing cell lines were plated 35 mm Petri dishes (10 cells/cm^2^) in 2 mL of medium and grown for 48 h at 37 °C. Cells were then irradiated in PBS containing Ca^2+^ and Mg^2+^ at 5, 10, 15 or 20 J/m^2^ of UVC radiation, and the preserved cell media replaced in its appropriated dish. Cells were grown for additional 12 days, and their media were changed every three-days. Then 500 µL of a crystal violet solution (0.5% *p*/*v*) in methanol/H_2_O 1:1 was applied for 10 min at room temperature to reveal the living cells. The dishes were then rinsed and the visible colonies counted.

### 4.4. Preparation of Frozen Pellets

Mock and APP751-transfected cell lines were plated in 75-cm^2^ flasks (Becton Dickinson Biosciences, Pont-de-Claix, France) and incubated for 48 h before treatment in the presence of G418. Each cell line was then treated with the amount of either H_2_O_2_ corresponding to the IC_10_ of the mock cell line by adding 1.5 mL of the 11X-concentrated solutions to the 15 mL of culture media already present in the flask. In control flasks, 1.5 mL of H_2_O alone was added. Cell lines were incubated for an additional 24 h and then collected by trypsinization, recovered, counted and pelleted by centrifugation at 300× *g* for 10 min. For UVC-associated experiments, Mock and APP751-expressing cells were plated in 100 mm petri dishes and incubated for 48 h before irradiation. Then, the culture medium was removed, kept aside and replaced by PBS containing Mg^2+^ and Ca^2+^. Cells were irradiated at 5 J/cm^2^ and either collected right after or the old culture media was added back to the petri dished to collect different kinetics points.

For both kinds of stress, the required amount of cells for each appropriate experiment was resuspended in FCS supplemented with 10% DMSO, gently frozen to −80 °C and stored in liquid nitrogen or dry-frozen.

### 4.5. DNA Extraction and Digestion

DNA extraction and digestion were performed as described previously [53]. Briefly, frozen cell pellets were resuspended in lysis buffer A (320 mM sucrose, 5 mM MgCl_2_, 10 mM Tris, 0.1 mM deferoxamine pH 7.5, 1% Triton X-100). The nuclei were then pelleted by centrifugation at 1500× *g* for 5 min and washed by 750 µL of buffer A. Then buffer B (10 mM Tris, 5 mM EDTA-Na_2_, 0.15 mM deferoxamine, pH 8.0) and SDS 10% was applied to allow the nuclei lysis. After adding a mixture of RNase A and RNase T1, the samples were incubated for 15 min at 56 °C. A second incubation step at 37 °C for 1 h with Qiagen protease was next performed. The subsequent additions of a solution of NaI (7.6 M NaI, 40 mM Tris, 20 mM EDTA-Na_2_, 0.3 mM deferoxamine, pH 8.0) and 100% isopropanol allowed DNA precipitation. Two washes were performed with first 40% isopropanol and then 70% EtOH. Finally, DNA was solubilised 0.1 mM deferroxiamine and DNA digestion was launched by a 2 h at 37 °C incubation with nuclease P1, phosphodiesterase II and buffer 10× (200 mM succinic acid, 100 mM CaCl_2_, pH 6.0) altogether. Then, a second digestion step was realized with phosphodiesterase I and alkaline phosphatase in alkaline phosphatase buffer 10× (500 mM Tris, 1 mM EDTA, pH 8.0) for 2 h at 37 °C. At last, the solution was neutralized with 0.1 mM HCl and centrifuged for 5 min at 5000× *g*. The supernatant was collected for analysis by HPLC-MS/MS.

### 4.6. Analysis of DNA Lesions (HPLC-MS/MS)

The digested sample was then injected onto an HPLC system consisting in a Agilent series 1100 system equipped with a Uptisphere ODB reverse phase column (2 × 250 mm ID, particle size 5 µm; Interchim, Montluçon, France). The mobile phase was a gradient of acetonitrile in a 2 mM aqueous solution of triethylammonium acetate. A UV detector set at 260 nm was used to quantify normal nucleosides in order to determine the amount of analyzed DNA. The HPLC flow was then directed toward an API 3000 electrospray triple quadrupole mass spectrometer operating in the negative ionization mode. The detection of TT-CPD, TC-CPD or CT-CPD was performed in the “multiple reaction monitoring” mode. Two fragmentations were monitored: 545 [M–H]^−^) → 447 [M–dehydrated 2-deoxyribose–H]^−^) and 545 [M–H]^−^) → 195 [phosphorylated 2-deoxyribose–H]^−^). External calibration was achieved for both detectors using authentic compounds.

### 4.7. Gene Expression Analysis

#### 4.7.1. mRNA Extraction and Quality Analysis

Total RNA was extracted from each sample with the GenElute mammalian total RNA miniprep kit (Sigma). RNA integrity was assessed verifying the A260/280 nm absorbance ratio between 1.7 and 2.1 and then using native gel electrophoresis. An amount of 500 ng per sample in native agarose gel loading buffer (15% ficoll, 0.25% xylene cyanol, 0.25% bromophenol blue) were loaded on 1% agarose gels in TBE (89 mM Tris-HCl pH 7.8, 89 mM borate, 2 mM EDTA) with 0.5 µg/mL ethidium bromide (Sigma) added to the gel. The gel was then run at 5–6 V/cm measured between the electrodes. Total RNA was considered as intact when 2 sharp 28S and 18S rRNA bands were visualized, with a 2:1 (28S:18S) ratio in term of intensity.

#### 4.7.2. Reverse Transcription

Total RNA was reverse transcribed, using Superscript II Reverse Transcriptase™ (Invitrogen) according to the manufacturer’s protocol. Briefly, 2 µg of total RNA were heat with 100 ng of Random Hexamers Primers (Promega SARL, Charbonnières, France) and 10 nmol of a dNTPs mix (Sigma) for 5 min at 70 °C and quick chilled on ice. Then 5X first-strand buffer to a final concentration of 1X, 0.1 M DTT and 45 U of ribonuclease inhibitor (Sigma) were added to the mixture and incubated for 2 min at 25 °C. 200 U of Superscript II Reverse Transcriptase™ were finally added to the mixture and incubated first for 10 min at 25 °C and next for 50 min at 42 °C. The reaction was stopped by heating for 15 min at 70 °C.

#### 4.7.3. Real Time Quantitative PCR

Gene specific Oligonucleotide primers (Table 1) were designed using Primer3-web 0.4.0 (http://primer3.sourceforge.net) and obtained from a commercial supplier (Eurogentec SARL, Angers, France). Quantitative PCR (qPCR) was performed in a MX3005p Multiplex Quantitative PCR system (Stratagene, La Jolla, CA, USA) using MESA Blue qPCR™ Mastermix Plus for SYBR^®^ Assay—Low Rox (Eurogentec, Angers, France). Quantitative PCR reactions were carried out in triplicate in 25 µL reaction volumes containing 200 nM forward and reverse primers and 20 ng template using the following program: 5 min at 95 °C, and 40 cycles of 15 s at 95 °C, 20 s at 55 °C and 40 s at 72 °C with fluorescence acquisition at the end of the 55 °C primer annealing step. The integrity of amplification indicated by a single melt peak for each product was verified by a dissociation curve analysis at 95 °C for 1 min, 55 °C for 30 s and 95 °C for 30 s with fluorescence acquisition at all points from 55 to 95 °C. The built in amplification-based proprietary algorithm (Stratagene) was used to set the fluorescence threshold value. This algorithm determines the portion of the amplification plots where all of the data curves display an exponential increase in fluorescence, and calculates the threshold value that minimizes the standard deviation in *C*_t_ values for each replicate set at a point which falls within 32.5% of the fluorescence shift for all the curves. The number of cycles (*C*_t_) at which the amplification-corrected normalized fluorescence (dRn) for each reaction crossed the threshold value was exported to Excel (Microsoft, Redmond, WA, USA) for further analysis.

**Table 1:** Primer sets used for real time quantitative PCR.

Name	GeneBank Entry	Forward Primer Sequence	Tm (°C)	Reverse Primer Sequence	Tm (°C)	Product Size (bp)
**XPA**	NM_000380.3	gcagccccaaagataattga	60.04	tggcaaatcaaagtggttca	60.09	183
**XPC**	NM_004628.4	ccatgaggacacacacaagg	60	tccaatgaaccacttcacca	59.94	187
**DDB1**	NM_001923.3	aacagagtggcgagagcatt	60.02	tcaatgacatgcagctcctc	59.95	223
**DDB2**	NM_000107.2	tcaaggacaaacccaccttc	59.94	gtgaccaccattcggctact	60	226
**XPD**	NM_000400.3	gctggacatctaccccaaga	60.07	ccggatcacagcaatatcct	59.92	166
**XPB**	NM_000122.1	gcggcagagattcttggtag	59.98	ggccccagacatagaactca	60.07	235
**ERCC1**	NM_202001.1	ttgtccaggtggatgtgaaa	59.94	gctggtttctgctcataggc	59.98	151
**PCNA**	NM_002592.2	ggctctagcctgacaaatgc	59.98	gcctccaacaccttcttgag	59.84	224

#### 4.7.4. Validation of Stably Expressed Housekeeping Genes

We tested 3 different housekeeping/reference genes in order to use them all for an optimal normalization of the target genes. S18, GAPDH and CycloB were amplified in triplicate in each sample and condition on a same qPCR run. Corresponding *C*_t_ were exported to BestKeeper [54], an Excel-based pair wise correlation tool that analyses variability in the expression in individual genes (SD and coefficient of variation) and generates a weighted expression index, the BestKeeper Index, in the form of a geometric mean of *C*_t_ values from several candidate reference genes. Then, correlation between each candidate and the index is calculated, describing the relation between the index and the contributing candidate reference gene by the Pearson correlation coefficient (*r*), coefficient of determination (*r*^2^) and the *p*-value. Individually S18, GAPDH and CycloB showed an SD smaller than 1. The analysis of Pearson coefficient correlation showed a strong correlation for all candidates. Individual SD and Pearson coefficient correlation of the 3 genes were tested in each PCR run and were always satisfying.

#### 4.7.5. mRNA Quantification and Statistical Analyses

Targeted genes’ mRNA expression was normalized to the expressed housekeeping genes S18, GAPDH and CycloB using Relative Expression Software Tool 2006 [55], which uses the pair-wise fixed reallocation randomization test as statistical models. Corresponding *p*-values were analyzed to evaluate the significance of expression ratio after each PCR run, but we used the Student’s *t*-test for comparing the mean of expression ratios between the two conditions after three to seven qPCR runs, equivalent to three to seven biological replicates, for each tested target gene.

### 4.8. DNA Repair Assays

The alkaline single-cell gel electrophoresis assay was used to measure DNA repair capacity of cell extracts, which involves slight changes of the precedent version. Briefly, frozen pellets of LnCap 10 J/cm^2^ UVC-irradiated were embedded in low-melt agarose, spread on microscope slides previously coated with one layer of normal agarose, gelled on ice and immersed in lysis solution overnight at 4 °C. The slides were neutralized and equilibrated in the Reaction buffer digestion buffer (3 times 5 min in 40 mM HEPES, 0.1 M KCl, 0.5 mM EDTA, 2 mg/mL BSA pH 8; Sigma). Frozen pellets of Mock and APP751-expressing cells treated or not by CuSO_4_ and H_2_O_2_ (3 × 10^6^ cells per condition) were centrifuged at 1400 rpm for 5 min at 4 °C, washed once in cold-PBS and resuspended in Extraction Buffer (45 mM HEPES, 0.4 M KCl, 1 mM EDTA, 10% Glycerol, 0.1 mM DTT, 0.25X Triton; Sigma). The mixture was then vortexed for 30 s, incubated for 5 min on ice and centrifuged at 14,000× *g* for 5 min at 4 °C. The supernatant was then added to a cold volume of Reaction Buffer (1:5). Incubation with these freshly prepared cell extracts (50 µL per slide) was performed for 30 min at 37 °C. Digestion with Fpg (1.7 µg/mL, 100 µL per slide) was used as positive controls and negative controls were mock-treated with a 1:5 mix of Extraction and Reaction Buffer. After digestion, the slides were transferred into the electrophoresis tank filled with electrophoresis buffer pre-chilled at 4 °C. The slides were left at room temperature for 30 min and electrophoresis was subsequently accomplished for 30 min at 25 V and 300 mA. After migration, the slides were rinsed in the neutralization buffer (5 min, room temperature) and stained with ethidium bromide.

We used the Comet Assay IV software (Perceptive Instruments, Suffolk, UK) to analyse the slides. Fifty randomly selected nuclei were scored by slide and triplicate slides were processed for each experimental point. The extent of damage was evaluated by the Tail DNA value defined as the percentage of DNA in the tail of the comet. Significance of differences between one sample and another was determined by Student’s *t*-test.

### 4.9. Cell Nuclear Extracts

Nuclear extracts were prepared as already described [56]. Briefly, thawed cells were washed twice in ice-cold PBS, suspended in 1 mL of ice-cold buffer A (10 mM HEPES pH 7.9, 1.5 mM·MgCl_2_, 10 mM·KCl, 0.01% Triton X-100, 0.5 mM DTT, 0.5 mM PMSF) and left on ice for 20 min. Cytoplasm membrane lysis was completed by vortexing the tube for 30 s. Lysis completion was controlled by trypan blue exclusion and nuclei were recovered by centrifugation 5 min at 5000 rpm at 4 °C. They were then suspended in 25 µL of ice-cold buffer B (10 mM HEPES pH 7.9, 1.5 mM MgCl_2_, 400 mM KCl, 0.2 mM EDTA, 25% glycerol, 0.5 mM DTT, anti-proteases (Complete-mini, Roche, Meylan, France) and 0.5 mM PMSF). Nuclear membranes were lysed for 20 min on ice, and two cycles of freezing-thawing at −80 and 4 °C respectively. The extracts were cleared by centrifugation for 10 min at 13,000 rpm at 4 °C. The supernatant was recovered and stored frozen in 10 µL aliquots at −80 °C. Protein content was determined using the BCA kit (Interchim, Montluçon, France). Typical protein content was 1 mg/mL.

## 5. Conclusions

In summary, we observed that, as for BER, the neurotoxic peptide Aβ interferes with NER. However, the effects are completely different. In a previous work using the same cellular model, we have shown that Aβ decreases BER activities and expression of a wide range of BER-associated genes, both at the basal level and in response to an oxidative stress. The present study reveals a drastically different impact of Aβ on NER. First, only two genes are affected by oxidative stress in the presence of Aβ, and in contrast to BER, are over expressed. Interestingly, the protein products of these two genes are also involved in the induction of apoptosis. This observation combined with the now well-established accumulation of DNA damage resulting from increasing oxidative insult and reduced BER repair, could explain why neurons easily undergo apoptosis in AD patients. Improving global DNA repair might therefore be a new strategy to prevent neuronal death in AD patients.
